# Mutation at the *Evi1* Locus in *Junbo* Mice Causes Susceptibility to Otitis Media

**DOI:** 10.1371/journal.pgen.0020149

**Published:** 2006-10-06

**Authors:** Nicholas Parkinson, Rachel E Hardisty-Hughes, Hilda Tateossian, Hsun-Tien Tsai, Debra Brooker, Sue Morse, Zuzanna Lalane, Francesca MacKenzie, Martin Fray, Pete Glenister, Anne-Marie Woodward, Sian Polley, Ivana Barbaric, Neil Dear, Tertius A Hough, A. Jackie Hunter, Michael T Cheeseman, Steve D. M Brown

**Affiliations:** 1 Mammalian Gentics Unit, Medical Research Council, Harwell, United Kingdom; 2 Department of Otorhinolaryngology, MacKay Memorial Hospital, Taipei, Taiwan; 3 Mammalian Genetics of Disease Group, School of Medicine, University of Sheffield, Sheffield, United Kingdom; 4 Mary Lyon Centre, Harwell, United Kingdom; 5 GlaxoSmithKline Pharmaceuticals, New Frontiers Science Park, Harlow, United Kingdom; Harvard Medical School, United States of America

## Abstract

Otitis media (OM), inflammation of the middle ear, remains the most common cause of hearing impairment in children. It is also the most common cause of surgery in children in the developed world. There is evidence from studies of the human population and mouse models that there is a significant genetic component predisposing to OM, yet nothing is known about the underlying genetic pathways involved in humans. We identified an *N*-ethyl-*N*-nitrosourea-induced dominant mouse mutant *Junbo* with hearing loss due to chronic suppurative OM and otorrhea. This develops from acute OM that arises spontaneously in the postnatal period, with the age of onset and early severity dependent on the microbiological status of the mice and their air quality. We have identified the causal mutation, a missense change in the C-terminal zinc finger region of the transcription factor *Evi1.* This protein is expressed in middle ear basal epithelial cells, fibroblasts, and neutrophil leukocytes at postnatal day 13 and 21 when inflammatory changes are underway. The identification and characterization of the *Junbo* mutant elaborates a novel role for *Evi1* in mammalian disease and implicates a new pathway in genetic predisposition to OM.

## Synopsis

Otitis media (OM), inflammation of the middle ear, is the most common cause of deafness in children. Although acute episodes of OM in children are associated with middle ear infections, in a substantial portion of cases recurrent episodes of OM, or a chronic suppurative OM, will develop. There is evidence from genetic studies of human families that there is a significant genetic component contributing to the development of recurrent and chronic forms of OM. However, the genes involved have not been identified. The authors have identified and characterized mouse mutants that demonstrate chronic OM as a route to identifying genes involved with OM. This study describes one mutant, *Junbo,* which shares many features with human OM. *Junbo* develops an acute OM following birth that subsequently develops into a chronic suppurative form of OM. *Junbo* carries a mutation in the transcription factor gene, *Evi1. Evi1* is expressed in a variety of cell types in the middle ear lining when inflammatory changes are underway. The identification of the *Junbo* mutation implicates a new gene involved in predisposition to OM.

## Introduction

Otitis media (OM), inflammation of the middle ear, remains the most common cause of hearing impairment in children [1,2]. Acute episodes of OM in infants and children are most often associated with middle ear infections involving the pathogens Streptococcus pneumoniae and Haemophilus influenzae [3]. Prolonged stimulation of the inflammatory response, along with poor mucociliary clearance, can also lead to the persistence of middle ear fluid giving rise to the clinical presentation of otitis media with effusion [2]. In a substantial portion of children, recurrent episodes of OM or a chronic suppurative OM will develop. The high prevalence of the disease, coupled with its recurrent and chronic nature, accounts for the large number of tympanostomies undertaken in affected children. OM is still the most common cause of surgery in children in the developed world. There is still considerable debate over the etiology of OM and the underlying pathological mechanisms [2]. However, risk factors for OM include craniofacial abnormalities, impaired mucocilliary function, and the presence of an inflammatory stimulus, such as bacteria. There is evidence from studies of the human population and mouse models that there is a significant genetic component predisposing to recurrent or chronic OM [4–7], yet little is known about the underlying genetic pathways involved. While several inbred strains are predisposed to the development of OM, their genetic analysis and utility is compounded by the complex genetic bases and the low penetrance of the phenotype [6]. In addition, there are several mouse mutants that demonstrate an OM phenotype, but the OM develops as part of a complex syndrome with a wide spectrum of phenotypes [6]. It will be important to identify and characterize the genes underlying highly penetrant mouse mutants that develop OM in the absence of other diverse pathology and represent appropriate models for OM in the human population.

Large-scale phenotype-driven mouse ENU (*N*-ethyl-*N*-nitrosourea) mutagenesis programs provide a rich source of novel mutant phenotypes that are the basis for systematic efforts to identify the genetic basis for diverse disease states [8–10]. One such screen at MRC Harwell, recovered a large number of mutant phenotypes representing ENU-induced mutations at a number of novel loci in the mammalian genome [9]. Mouse models have and continue to play an important role in studying the genetic causes of hearing impairment. A number of mutations have been cloned and have provided us with several profound insights into the critical proteins involved with the development and function of the auditory apparatus at the level of both the middle and inner ears [11,12]. Nevertheless, it is clear that we do not possess mouse mutants for all loci and pathways potentially involved in hearing impairment.

We report the characterization of a new mutant, *Junbo,* identified in the Harwell mutagenesis program as having a deafness phenotype. *Junbo* is a model of OM that shares many features with the human condition. Acute OM arises spontaneously in the postnatal period and develops into chronic suppurative OM with otorrhea. The underlying molecular basis of this phenotype has been identified as a mutation in the *Evi1* transcription factor causing a nonconservative Asn763Ile change in the second of the two zinc-finger domains in this protein. The *Junbo* mouse highlights a new role for the transcription factor *Evi1* and provides the first evidence for the genetic pathways involved in the etiology of this complex childhood disease.

## Results

### Identification, Mapping, and Cloning of the *Junbo* Mutation

**Figure 1:**
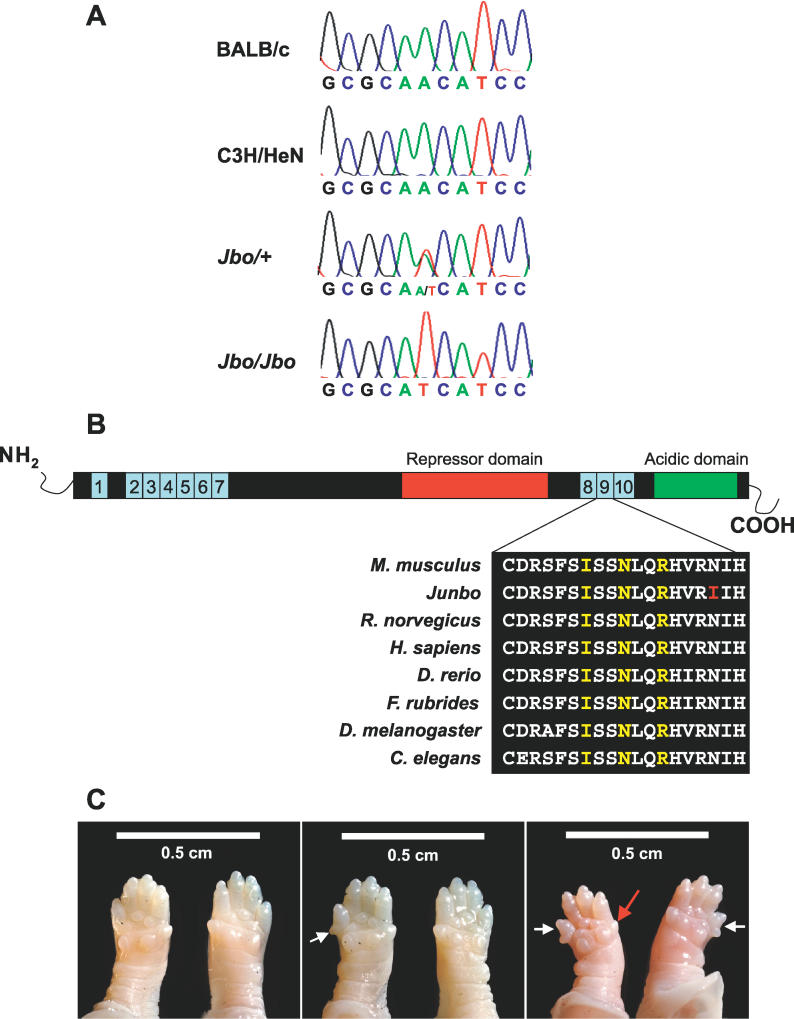
The *Evi1* Gene Is Mutated in *Junbo* Mice (a) Sequence analysis of the *Evi1* locus in BALB/c, C3H/HeN, *Jbo*/+ adult, and *Jbo/Jbo* embryonic DNA. An A2318T transversion is detected in *Jbo/+* and *Jbo/Jbo* mutants that is not present in either parental substrains. (b) Schematic of the EVI1 peptide. Ten zinc finger motifs are clustered into two DNA-binding domains, ZF1 and ZF2. EVI1 contains a proline-rich repressor domain between the two sets of zinc fingers and a highly acidic domain at the C-terminus. Expanded peptide sequence across the ninth zinc finger motif shows the high degree of conservation of this region between orthologous proteins from different species: Mus musculus (P14404), Rattus norvegicus (ENSRNOG00000012645), Homo sapien (Q03112), Danio rerio (ENSDARP00000008993), Fugu rubrides (ENSDARP00000008993), Drosophila melanogaster (CG31753), Caenorhabditis elegans (R53.3a). Contact residues are highlighted in yellow, the position of the *Junbo* mutation is highlighted in red. (c) Extra digits are seen on the forelimbs of both heterozygote and homozygote mice E18.5 (white arrows). In heterozygotes, (*Jbo/+*, middle panel) an extra digit is observed on either forelimb. The homozygotes, (*Jbo/Jbo,* right panel) have extra digits on both forelimbs. The anterior digit is often reduced in size in the homozygote limbs (red arrow). Wild-type mice, left panel.

An ENU mutagenesis screen [9] identified a new dominant mutant, *Junbo (Jbo),* with hearing loss. Preliminary phenotyping using a click-box test (see Materials and Methods) of an age-matched cohort derived from the founder mouse indicated that mice demonstrated a hearing loss at ~40 d after birth (DAB). We used a pooling strategy employing DNA from affected mice and genome-wide fluorescent simple sequence length polymorphism-based screening [9,13] to provide an initial map position for the *Jbo* mutation on Chromosome 3. This map position was further refined using additional affected animals to an approximately 1.5-Mb region delineated by the *Eif5a2* locus and microsatellite marker *D3Mit178.* Direct sequence analysis of coding regions within this interval identified an A2288T transversion in the *Evi1* locus, causing a nonconservative Asn763Ile change in the second of the two zinc-finger domains in this protein (Figure 1A and 1B). No other sequence changes were identified in any other coding sequences within the minimal nonrecombinant region containing the mutation.

A knockout allele of *Evi1* has been produced, *Evi1^tm1Mmor^* [14]. *Evi1^tm1Mmor^* mice carry a targeted deletion resulting in an isoform-specific null for the longest *Evi1* transcripts, while the Δ324 (shorter) isoform [15,16] remains unaltered. This isoform lacks the final two zinc-finger motifs from the first (N-terminal) DNA-binding domain and consequently is predicted to exhibit altered binding abilities. Characterization of heterozygote mice carrying this targeted mutant failed to uncover any phenotype, while homozygotes show prenatal lethality from presumptive cardiac failure [14]. In addition, these embryos displayed widespread hypocellularity, most markedly of the cortical mesenchyme, retarded development of the first and second branchial arches, and severely reduced vasculature of the yolk sac. To confirm that the A2318T alteration identified in *Evi1* is responsible for the *Junbo* phenotype, we undertook a complementation screen between *Jbo*/+ and *Evi1^tm1Mmor^*/+ animals. 81 live births were produced from crosses of *Jbo*/+ and *Evi1^tm1Mmor^*/+ mice and all were genotyped. None of the progeny coinherited the *Evi1^tm1Mmor^* and the *Jbo* mutations. However, the other expected genotypes were recovered—*Jbo*/+ (38%), *Evi1^tm1Mmor^*/+ (28%), +/+ (33%)—confirming the allelism of the knockout and *Junbo* mutant. We established intercrosses between *Jbo/+* animals using in vitro fertilization and implantation into wild-type females as *Jbo/+* females undergo repeated spontaneous abortion. At 10.5 days postcoitum (dpc), 17% of homozygote mice had small hind limbs, a large pericardial sac, and a malformed forebrain, features demonstrated by the knockout [14]. However, many homozygotes were scored as normal in appearance at this and later stages and proceeded to die between E18.5 and birth, though homozygotes were of comparable body size to wild-type littermates. It appears that though some homozygote embryos may die at 10.5 dpc, others continue to develop unhindered when carried by a wild-type mother. The only other phenotypic feature detected in *Jbo/+* mice was an extra digit on one forelimb. *Jbo/Jbo* mice have extra digits on both forelimbs (see Figure 1C).

### Pathology Phenotyping of the *Junbo* Mutant

To determine the cause of the deafness phenotype in *Junbo,* we performed an initial examination of deaf adult animals and embryonic skeletal preparations (15.5 dpc–18.5 dpc). This revealed no gross morphological defects of the inner ear and ossicular chain or ossification of the temporal bone and tympanic ring in *Jbo/+* animals. Dissection of the bullae of seven *Jbo/+* mice and four *+/+* mice (>130 DAB) revealed that the middle ear cavity (MEC) of mutant mice was filled with exudate, indicative of OM. X-ray analysis revealed that there was no consistent abnormality of bullae shape associated with OM in *Jbo/+* mice. Examination of seven *Jbo/+* mice (14 bullae, >180 DAB) revealed that 6 *Jbo/+* mice bullae had a normal shape and demonstrated radio opacity, indicating the presence of an effusion in the middle ear, whereas in six *Jbo/+* mice, bullae were misshapen and also had an effusion present (demonstrated by radio opacity). Although X-ray imaging is a less sensitive means of diagnosing OM than histology, two *Jbo/+* mice bullae did not demonstrate a radio opacity reflecting the possibility of resolution of a small number of *Junbo* mice at advanced age (see below). Control ears (two wild-type mice, four bullae) were clear of effusion and presented with a normal bullae shape. We concluded that the presence of abnormally shaped bullae in the *Junbo* mutant is not necessary for the development of an effusion. We therefore proceeded to study the pathology of the OM in the postnatal period, at weaning, and in the adult, first in conventionally housed mice of relatively low microbiological status and then in specific pathogen-free (SPF) mice.

**Figure 2:**
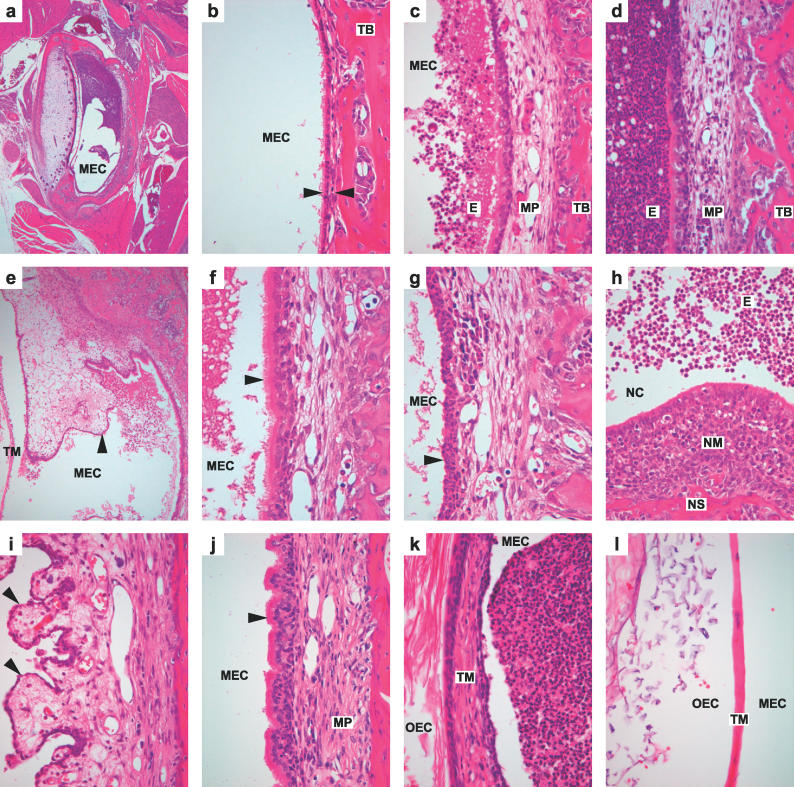
Histology of Middle Ear and Nose in Wild-Type and *Junbo* Mutant Mice Images (a–h) are from 13-d-old postnatal mice and are given with their original magnification (a) *Jbo/+* dorsal section of MEC partly filled with exudate ×40, (b) +/+ normal middle ear temporal bone covered with thin mucoperiosteum (arrowheads) ×400, (c) +/+ inflamed middle ear with thickened mucoperiosteum with neutrophil leukocyte infiltrates and neutrophil-rich exudates in the MEC ×400, (d) *Jbo/+* middle ear with more severe suppurative exudation into the MEC ×400, (e) *Jbo/+* inflamed edematous polyp (arrowhead) of un-resorbed embryonic middle ear connective tissue, tympanic membrane ×100, (f) *Jbo/+* MEC lined by ciliated columnar cells (arrowhead) ×600 (g) *Jbo/+* MEC lined by basal cells (arrowhead) ×600, (h) *Jbo/+* suppurative rhinitis: nasal cavity with suppurative exudate, nasal septum with inflamed nasal mucosa ×200. Images (i–l) are of adult (180-d) *Jbo/+* middle ear with chronic suppurative OM; changes include (i) fibrous polyps (arrowheads) ×200, (j) hyperplasia of ciliated epithelial cells (arrowhead) and fibrosis of mucoperiosteum stroma ×400, and (k) fibrous thickening of the tympanic membrane, outer ear canal ×400 compared with (l) normal +/+ tympanic membrane, outer ear canal ×400. E, exudate; MP, mucoperiosteum; NC, nasal cavity; NM, nasal mucosa; NS, nasal septum; OEC, outer ear canal; TB, temporal bone; TM, tympanic membrane

**Figure 3:**
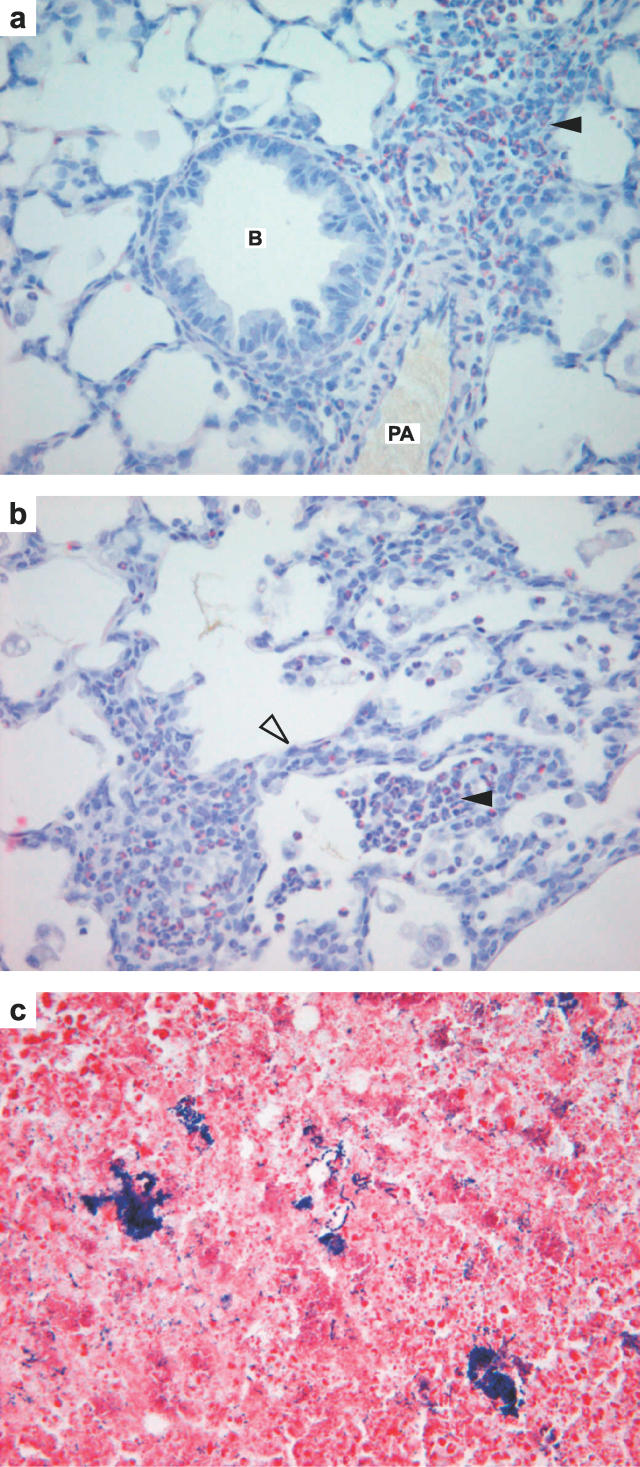
Histology of the Lung and Middle Ear Exudate in *Junbo* Mice (a) 13-d postnatal *Jbo/+* lung with perivascular and peribronchiolar cuffs containing Sirius red positive eosinophil leukocytes (arrowhead), bronchiole, pulmonary artery ×400, (b) focal eosiniphilic alveolitis (arrowhead) and thickened alveolar septae (open arrowhead) with eosinophil-rich infiltrates. (c) 21-d postnatal *Jbo/+* MEC pus with colonies of Gram positive cocci ×600. B, bronchiole; PA, pulmonary artery

In conventionally housed mice we found no evidence of inflammation of the embryonic connective tissue or exudation into the MEC in *Jbo/+* or wild-type litter mates at 5 DAB. By 13 DAB the MEC is more fully formed and 100% of the *Jbo/+* mice and ~33% wild-type mice had acute OM (Figure 2A–2D). However, *Jbo/+* mice had suppurative exudate in both the MECs, while wild-type mice had unilateral OM, or serous exudation in one ear and suppurative exudation in the contralateral ear. Typical acute inflammatory changes in the MEC mucoperiosteum included edematous polyps (Figure 2E, which may represent unresorbed embryonic connective tissue [17]) and/or bulging sub-epithelial bullae filled with serous fluid, stromal edema, widely patent capillaries, and lymphatics, with variable numbers of infiltrating neutrophil leukocytes. The epithelial covering was formed by small basal cells or ciliated columnar cells (Figure 2F and 2G). A second, milder type of OM occurred in a further ~27% of wild-type mice consisting of only focal mucoperiosteal aggregations of neutrophil leucocytes with or without a light neutrophil leukocyte MEC exudation (unpublished data); ~40% wild-type mice had no evidence of OM at this age. Importantly, OM appeared at this stage as part of a more generalized respiratory tract inflammation comprising suppurative rhinitis (Figure 2H) and nasopharyngitis, and multifocal mild alveolitis/interstitial pneumonia with eosinophil leukocytes in perivascular cuffs (Figure 3A and 3B) but without evidence of intralesional bacteria or viral inclusions. There were no significant histological lesions in intestine, liver, pancreas, kidney, heart, thymus, and spleen.

Any postnatal OM had resolved in wild-type mice by weaning (0% incidence at 21 DAB) and OM was exceptional in adults (3% incidence). In contrast, OM was present in 100% of *Jbo/+* mice at weaning and occurred in 94% of adult *Jbo/+* mice 29 DAB to >180 DAB. Suppurative rhinitis was still present in some *Jbo/+* mice examined, but in two the eosinophilic pneumonia was absent or minimal. Large numbers of Gram positive cocci were present in OM exudate at day 21 in ~70% of cases (Figure 3C), suggesting nasopharyngeal staphylococcal and streptococcal flora may play a role in progression of OM, if not its initiation in *Jbo/+* mice. There was no evidence of significant proliferation of mucous cells or periodic acid-Schiff-positive mucus in the MEC.

Adult *Jbo/+* mice >29 DAB develop bilateral chronic suppurative OM. Chronic middle ear effusions contained variable numbers and proportions of viable and necrotic neutrophil leukocytes and foamy macrophages; bacteria were infrequently present and there were small numbers of multinucleate macrophages, cholesterol clefts, and small amounts of birefringent foreign body (perhaps secondary to perforation of the eardrum). The effusions did not contain significant amounts of periodic acid-Schiff-positive mucus. Chronic changes in inflamed thickened mucoperiosteum include formation of multiple polyps (Figure 2I) covered by low cuboidal cells or hyperplastic ciliated columnar cells and scattered mucous cells (Figure 2J). The mucoperiosteal stroma has variable degrees of fibrosis, neutrophil leukocyte infiltration, scattered mast cells, lymphoplasmacytic infiltrates, and occasional lymphoid nodules. The eustachian tube is patent and can contain exudates, and the epithelial lining can have elevated numbers of mucous cells and intra-epithelial leukocytes. Fibrous thickening of the eardrum (tympanosclerosis) is common (Figure 2K and 2L). OM was often associated with perforation of the eardrum, possibly providing drainage to account for apparent resolution of a few cases: (6%) of OM in the oldest age group of *Jbo/+* mice (>180 DAB).

The *Junbo* colony has subsequently been re-derived by embryo transfer into new high-health status facilities (Mary Lyon Centre, Harwell). Mice are housed in individually ventilated cages, and screening shows all FELASA-listed pathogens (see Materials and Methods) have been excluded; 75 cage air changes/h reduce respiratory irritants such as ammonia to <3 ppm. In SPF conditions, OM in *Jbo/+* mice is relatively milder at early time points and is not associated with rhinitis. Typically at 13 DAB there are small numbers of inflammatory cells in the mucoperiosteum and sometimes, light MEC effusion. By 20–22 DAB and 28 DAB, 4% *Jbo/+* mice had bilateral OM, 54% unilateral OM, and 42% had very mild or no OM. However, by 54 DAB, *Jbo/+* mice (100%) had OM, but in 10% of cases this was unilateral. OM does not occur in pre-weaned wild-type mice and in only 3% (a single case in a 22-DAB mouse) of older wild-type mice.

Comprehensive pathology phenotyping failed to show a consistent pattern of significant organ pathology outside of the middle ear in adult *Jbo/*+ mice. Specifically there is no evidence of opportunistic infections in sites such as skin, lung, urogenital, or gastrointestinal systems that might be a sign of immune deficiency (see below).

In summary, the microbiological status of the mice and/or air quality affect onset of OM, and under “dirty” conventional conditions even wild-type mice can develop OM in the postnatal period as part of upper respiratory tract disease; however, this resolves in wild-type mice by weaning. In conventionally housed and SPF *Jbo/+* mice, OM emerges as a chronic condition.

### Immunology and FACS Analysis of SPF Wild-Type and *Junbo* Mice

There were no significant differences in the antibody responses of *Jbo/+* and wild-type control mice to immunization with T-dependent (IgG1 and IgG2a to keyhole limpet hemocyanin) and T-independent (IgGM and IgG3 to pneumococcal polysaccharide type 3) antigens (Table S1). FACS analysis of blood neutrophils identified by the cell surface markers Gr-1 and Mac-1 did not reveal significant differences between levels of immature, mature cell forms, and total circulating blood neutrophils in *Jbo/+* and wild-type mice at either 20–22 DAB or 49–58 DAB. However, there was a significantly lower (*p* = 0.003) ratio of immature forms in the circulating neutrophil pool in 20–22 DAB *Jbo/+* mice, but not in 54–58 DAB *Jbo/+* mice (Table 1).

**Table 1:**
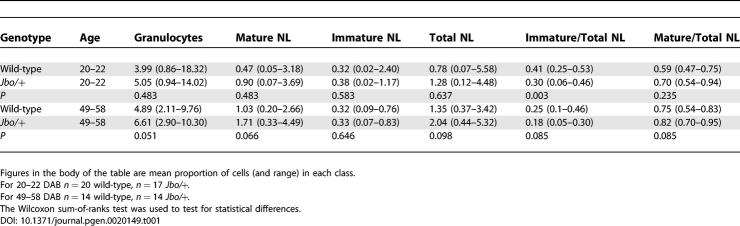
FACS Analysis of the Proportion of Granulocytes in Blood of Wild-Type and *Junbo* Mice

### Expression of *Evi1* in Wild-Type and *Junbo* Mice

**Figure 4:**
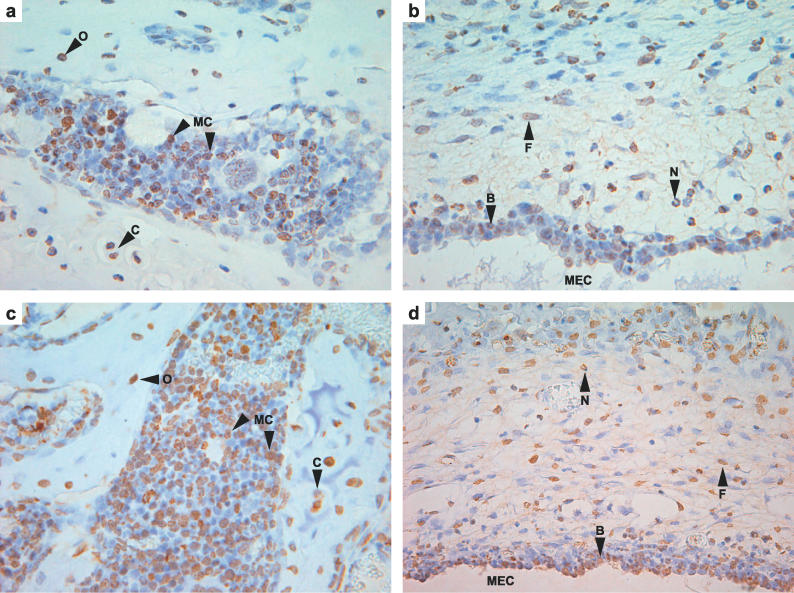
Evi1 Protein Immunostaining in 13-d-Old *Jbo/+* and Wild-Type Mice with Acute OM (a) *Jbo/+* positive myeloid cells in temporal bone marrow ×600. Note chondrocytes and osteocytes are also strongly positive. (b) *Jbo/+* mucoperiosteum has positive neutrophil leukocytes, fibroblasts, and basal epithelial cell nuclei ×600. (c) *+/+* similar pattern of staining in bone marrow ×600 and (d) +/+ inflamed mucoperiosteum ×600. B, basal epithelial cell nuclei; C, chrondrocyte; F, fibroblast; MC, myeloid cell; N, neutrophil leukocyte; O, osteocyte

We proceeded to investigate the expression of *Evi1* in both embryonic whole-body tissues (E9.5 and at eight intervals through to birth) and postnatal middle ear tissue (13 DAB, 21 DAB) in order to relate the underlying mutation to the *Junbo* phenotype. We examined expression in wild-type and *Jbo/+* mice, but at no time point did we identify any significant differences in expression patterns, up to and including birth, from those described [14,18]. However, in postnatal head tissues, we now find that *Evi1* is expressed in nuclei of myeloid cells in bone marrow, neutrophil leukocytes, fibroblasts, and basal epithelial cells in the inflamed middle ear lining (Figure 4), and that patterns of expression are similar in *Jbo/+* and wild-type mice.

### OM Phenotype of the *Evi1^tm1Mmor^/+* Mice

OM is not a prominent feature in *Evi1^tm1Mmor^*/+ mice. Only one of 12 *Evi1^tm1Mmor^*/+ mice >68 DAB had OM, and in this single case it was unilateral. OM was not present in 15 wild-type littermates. The absence of OM in *Evi1^tm1Mmor^*/+ mice, coupled with the presence of extra digits in *Jbo/+* and *Jbo/Jbo* mice, suggests that the *Junbo* mutation may have gain-of-function effects. However, the genetic background of *Evi1^tm1Mmor^*/+ mice differs from *Jbo/+* and *Jbo/Jbo* mice (see Materials and Methods). In addition, the *Evi1^tm1Mmor^* mutation is an isoform- specific null. It is therefore difficult to reach definitive conclusions as to the nature of the *Junbo* mutation (see Discussion).

## Discussion

A mutation in the *Evi1* transcription factor underlies the development of a chronic suppurative OM in the *Junbo* mutant. Genetic mechanisms appear to interact with microbiological status and environmental conditions, such that SPF *Jbo/+* mice with improved air quality have milder early OM. Gnotobiotic studies are planned to investigate the role of nasopharyngeal *Staphylococcus* and Streptococcus spp. commensals in OM pathogenesis. The absence of multi-systemic inflammation and opportunistic infections at other sites, and the normal T-dependent and T-independent immune responses in *Jbo/+* mice, argue against an overt immune deficiency being responsible for OM.

The *Evi1* locus was initially identified as a common site of retroviral integration underlying susceptibility to myeloid tumors in the AKXD mouse recombinant inbred strain [19,20]. Transcriptional activation of the *Evi1* locus by translocations and inversions leads to myeloid leukemias and myelodysplastic syndrome in humans [21]. This locus encodes a 145-kDa nuclear transcription factor with two distinct zinc-finger domains composed of seven and three zinc-finger motifs, respectively [22]. Each zinc-finger domain has been shown to bind specific target consensus DNA sequences, and additional proline-rich and highly acidic domains located within the protein have been demonstrated to be capable of repressing or activating target promoter activities, respectively [21–24]. *Evi1* has also been shown to be capable of repressing the TGF-β signaling pathway through direct binding of Smad3 mediated by the first zinc-finger domain, suggesting a functional role in the control of cell development and proliferation [25]. In addition, cell line assays have demonstrated a role for *Evi1* in the transcriptional control of *c-fos* and the AP-1 proliferative pathway through interactions of the second zinc-finger domain [26]. The *Junbo* mutation results in a nonconservative Asn763Ile change in the second of these three zinc-fingers. Previous in vitro site-directed mutagenesis studies of the contact residues within these three zinc-fingers uncovered a complete loss of DNA-binding ability following any residue change [23]. The *Junbo* Asn763Ile alteration is within three amino acids of a contact residue, and the mutated amino acid contributes to the putative alpha helix of the Cys_2_His_2_ structure. While it seems likely that the *Junbo* mutation will disturb the function of the second zinc-finger domain, it is unclear whether or not there are effects on the role of the first zinc-finger domain that is involved in TGF-β signaling.

It is interesting to note that OM is not part of the phenotype of *Evi1^tm1Mmor^*/+ mice. However, the *Evi1^tm1Mmor^* allele results in an isoform-specific allele for the longest Evi1 transcript while the Δ324 shorter isoform is unaffected. In addition, the *Evi1^tm1Mmor^* knockout was established in ES-D3 cells with chimaeras subsequently mated to CF-1 mice [14]. Both the presence of the shorter isoform and the dissimilar genetic backgrounds between *Evi1^tm1Mmor^*/+ mice and *Jbo/+* mice may contribute to the differences in the expressivity of the OM phenotype. Alternatively, the *Junbo* mutation may lead to gain-of-function effects. However, it is not possible at this stage to distinguish between these possibilities.

A mutation in the *Evi1* transcription factor may give rise to OM by more than one mechanism, given that it is expressed in a number of different cell types in the middle ear. One mechanism arises from our observation that *Evi1* is expressed in neutrophil leukocytes during OM development**.** *Evi1* has multiple functions relating to hematopoietic differentiation and development of myeloid leukemia [27–30]. One putative target gene for *Evi1* in neutrophil leukocytes is the inositol triphosphate type 2 receptor gene *(Itpr2)* that is required for functional regulation of neutrophil leukocyte maturation via F-met-leu-phe receptor signaling in response to bacterial proteins [31]. Our FACS analysis of circulating neutrophils at two time points, when OM first appears and when chronic suppurative OM is well established, indicates that localized inflammation in the middle ear in *Jbo/+* mice does not result in significant neutrophilia. Neutrophils are apparently released from hematopoietic tissues at comparable levels in *Jbo/+* and wild-type mice and there was no detectable block in neutrophil development in *Jbo/+* mice. The ratio of immature neutrophils in the circulating pool was no higher; indeed, it is reduced in recently weaned mice when ~60% have acute OM. In older *Jbo/+* mice with fully penetrant chronic suppurative OM, immature and mature neutrophil ratios are not significantly different from wild-type mice.

A variety of in vitro studies have highlighted the role of the TGFβ/SMAD pathway on mucin expression and thus underlined the potential importance of this signaling pathway in OM [32]. A loss of *Evi1* function could affect TGFβ/SMAD signaling pathways. There are two in vitro studies that suggest that *Evi1* mutations could affect mucin expression that might underlie predisoposition to OM. Firstly, nontypeable Haemophilus influenzae (NTHi), a known bacterial pathogen involved in human OM, activates Tgfβ receptor-Smad3/4 signaling that together with TLR2-MyD88-TAK1-NIK-IKKβ/γ-IκBα-dependent activation of NF-κB is known to mediate NTHi-induced MUC2 mucin transcription [33]. *Evi1* loss-of-function mutations might lead to a de-repression of the TGFβ/SMAD pathway and an upregulation of MUC2 expression leading to an enhancement of effusive processes as a contributor to OM. Alternatively, it has also been shown that NTHi upregulates MUC5AC mucin production via activation of the TLR2-MyD88-dependant p38 pathway [34]. However, the activation of TGFβ/SMAD signaling by NTHi also leads to down-regulation of p38 activity by inducing MAPK phosphatase-1 and suppressing MUC5AC mucin induction. Thus, in this case loss of *Evi1* function and de-repression of the TGFβ/SMAD pathway would presumably lead to increased suppression of MUC5AC mucin induction that may reduce mucociliary defense in suppurative OM. Importantly, the identification of the *Junbo* mutation now provides in vivo evidence to support the role of these signaling pathways in OM. It will be interesting to explore further the molecular phenotype of the *Junbo* mutant and to examine whether disregulation of mucin expression is a contributing factor in the development of OM.

Additional levels of complexity of *Evi1* function relevant to OM pathogenesis may result from the first (N-terminal) zinc-finger domain binding to a number of putative target genes *Gadd45g, Gata2, Zfpm2*/*Fog2, Skil* (SnoN), *Klf5* (BTEB2), *Dcn,* and *Map3k14* (Nik) [35]. For instance, *Evi1* regulation of Nik could act in the TLR2-MyD88-TAK1-NIK-IKKβ/γ-IκBα-dependent activation of NF-κB pathway to mediate NTHi-induced MUC2 mucin transcription as described above; and also in the pro inflammatory cytokine IL-1 signaling pathway IL-1R1-MyD88-IRAK-TRAF6-TAK1-NIK-IKK-NF-κB/IκB [36].

In conclusion, the *Junbo* mutant provides an important genetic disease model of OM; particularly because it shares important features with the chronic human condition [7]. Inflammatory disease is restricted to the middle ear and is not a consequence of overt immune deficiency. OM arises spontaneously in the postnatal period, develops into chronic suppurative OM with otorrhea, with the early severity and age of onset dependent on microbiological status and/or air quality. We have shown that a mutation at the *Evi1* locus underlies the susceptibility and persistence of OM. Our observations underline the role of *Evi1* in mucin gene regulation as a possible contributor to OM. In this regard, the *Evi1* gene and associated pathway members can be considered important candidates for examining the genetic basis for susceptibility to OM in the human population.

## Materials and Methods

### Mice and deafness screening.

The founder mouse carrying the *Junbo* mutation was generated in a large-scale ENU mutagenesis program at Harwell, United Kingdom [9]. Male BALB/c mice were mutagenized and mated to normal C3H/HeN females, and the offspring were screened for a variety of defects, including deafness and vestibular dysfunction. The *Jbo* founder was identified because of a lack of a Preyer reflex when presented with a calibrated 20 kHz 90 dB SPL tone burst. For analysis of the phenotype, the colony was maintained on a C3H/HeN background in accordance with Home Office regulations.

### Microbiological status of the mice*.*

*Junbo* mice were originally derived in a conventional facility. Sentinel mice from this colony were seropositive for the FELASA- [37] listed viral agents MHV (judged by histology to be enteropathic strains), Adenovirus II, and TMEV, none of which are primary respiratory pathogens [38]. Intestinal flagellates, pinworms, and the opportunist respiratory pathogen Pasteurella pneumotropica were also common isolates. A number of pathogens known to cause respiratory disease and/or OM in the mouse such as pneumonia virus, Sendai virus, *Mycoplasma pulmonis, Streptococcus pneumoniae,* and *Pseudomonas aeruginosa* have not been found over many years in the sentinel screens. Non-FELASA-listed bacteria isolated from the nasopharynx of sentinel mice include Staphylococcus spp., *Staphylococcus aureus,* Alpha-haemolytic streptococci, and other Streptococcus spp. *Junbo* mice are now housed in a high- health-status SPF unit in which all FELASA-listed pathogens have been excluded. Non-FELASA nasopharyngeal flora remains the same.

### Genetic crosses and mapping.

The founder C3HeNBALB/c*^ENU^*F1-*Jbo/+* mouse was maintained by repeated backcrossing to C3H/HeN. Mutant progeny were identified by the lack of a Preyer reflex. 41 DNAs were initially pooled from affected individuals and a genome scan was carried out as described [9,13]. A high-resolution genetic map was constructed using 242 affected progeny N4 and N5 backcross progeny using published microsatellite markers *D3Mit90, D3Mit328, D3Mit92, D3Mit203, D3Mit55, D3Mit178, D3Mit151, D3Mit273, D3Mit180, D3Mit239, D3Mit21, D3Mit224, D3Mit182, D3Mit119, D3Mit241,* and *D3Mit22.* The genetic map across the *Jbo* locus was constructed by minimizing the number of recombination events across the region.

The *Evi1^tm1Mmor^* mutation was generated in D3 ES cells with subsequent matings to CF-1 mice [14]. *Evi1^tm1Mmor^*/+ mice were mated to C3H/HeN mice before crossing to *Jbo/+* mice for complementation testing. For phenotypic analysis *Evi1^tm1Mmor^*/+ mice were also mated to C3H/HeN mice and F1 heterozygous mutant progeny along with wild-type littermate controls aged to appropriate time points.

### Pathology, histology, and X-Ray analysis.

The onset and time course of development of the middle ear disease was examined by histology in conventionally housed postnatal mice 4–5 DAB (five *Jbo/+*, two +/+); postnatal 13 DAB (13 *Jbo/+*, 16 +/+); at weaning 21 DAB (seven *Jbo/+*, 17 +/+); and in adult mice 29 DAB (five *Jbo/+*, 11 +/+), 44 DAB (six *Jbo/+*, eight +/+), 180 DAB (seven *Jbo/+*, nine +/+), and 180–360 DAB (six *Jbo/+*, three +/+). Representative mice from one to three litters were examined at each time point. In a second study to assess the OM phenotype under SPF conditions, *Junbo* mice were sampled from an embryo re-derived colony that was housed in individually ventilated cages. The cohorts were as follows: 5 DAB (four *Jbo/+*, nine +/+), 13 DAB (11 *Jbo/+*, 19 +/+), 20–22 DAB, 28 DAB (24 *Jbo/+*, 17 +/+), 49–58 DAB (20 *Jbo/+*, 18 +/+), and 85–115 DAB (19 *Jbo/+*). 12 conventionally housed 68-DAB *Evi1^tm1Mmor^*/+ mice and 15 same-aged wild-type littermates were also examined for OM.

Tissues were fixed 24–48 h in 10% neutral buffered formalin (NBF) and embedded in paraffin wax. Heads and bones were decalcified 24–48 h with Immunocal (Decal Corporation, Tallman, New York, United States). 4-μm dorsal plane sections of decalcified middle ears were stained with Haemotoxylin and Eosin (H & E). In 13- and 21-DAB mice with OM, transverse sections of the nasal turbinates, dorsal plane sections of the oropharynx, lungs, livers, kidneys, heart, spleen, intestines, pancreas, and livers were examined by histology. Sections of middle ears, snouts, and lungs from OM cases were examined for bacteria using Gram stain and Sirius red for eosinophil leukocytes.

To assess the possibility of opportunistic infections at other body sites, a general pathology screen was performed on four 28-DAB and seven 56-DAB SPF *Jbo/+* mice. In addition, a more extensive whole-body pathology analysis of 25+ tissues following EMPReSS necropsy SOPs [39] was performed on four female and three male 180-DAB *Jbo/+* mice; five female and four male wild-type litter mates were used as controls.

For X-ray analysis, heads were skinned and the brains and mandibles removed, and then the skull was fixed in 10% NBF. After the dorsoventral view was taken, the head was bisected midsagittally and lateromedial views taken. Images were taken on a MX-20 Faxitron X-ray machine (Faxitron X-ray Corporation, Wheeling, Illinois, United States) at 26 kV and 0.3 mA with an exposure time of 3 s.

### Immunohistochemistry.

Whole embryos (E9.5, E10.5, E11.5, E12.5, E13.5, E16.5, and E18.5) and adult heads (13 DAB and 21 DAB) from wild-type and *Jbo/+* mice were used for immunolabelling. 3-μm wax sections were de-paraffinised in xylene substitute and rehydrated through graded ethanol solutions. Endogenous peroxidase activity was quenched with 3% hydrogen peroxide in isopropanol. The sections were microwaved in 10 mM citrate buffer (pH6.0) and rinsed with phosphate-buffered saline at room temperature. The immunostaining was performed using a Dako (Glostrup, Denmark) autostainer at room temperature. To inhibit the non-specific endogenous biotin staining the Dako Biotin Blocking System was used. A blocking solution of 10% donkey serum (Serotec) was used for 1 h. Goat polyclonal antibody raised against the carboxy terminus of human EVI1 was used in concentration 1:100 (Santa Cruz Biotechnology, Santa Cruz, California, United States) for 1 h. Biotinylated donkey anti-goat antibody (Santa Cruz Biotechnology) and ChemMate Detection Kit (Dako) were used to develop the specific EVI-1 signals. Negative control sections were incubated in donkey serum and processed identically. The slides were counterstained with Haematoxylin.

### Skeletal preparations.

Embryos were fixed in 95% ethanol and processed through a standard alcian blue and alizarin red bone/cartilage staining procedure.

### Immunology and FACS analysis in SPF mice.

The T-dependent arm of the immune system was assessed by immunization with keyhole limpet hemocycanin and measurement of IgG1 and IgG2a, and the T-independent arm by immunization with pneumococcal polysaccharide type 3 and measurement of IgGM and IgG3 in 42–56-DAB SPF mice (ten *Jbo/+*, ten +/+) [40]. For FACS analysis whole blood was collected in lithium heparin tubes from the jugular vein after overdosing mice with barbiturate-administered IP. SPF mice were assessed at 20–22 DAB (17 *Jbo/+*, 20 +/+) and 49–58 DAB (14 *Jbo/+*, 14 +/+). FACS analysis of granulocytes was performed after labeling cells with R-PE-labeled Gr-1 and FITC-labeled Mac-1 markers. The Wilcoxon sum-of-ranks test was used to test for statistical differences between *Jbo/+* and +/+ post-bleed antibody titers and granulocyte parameters.

## Supporting Information

Table S1 T-Dependent and T-Independent Responses in Immune- Challenged Wild-Type and *Junbo* Mice (35 KB DOC)

## Abbreviations

DAB: days after birth
dpc: days postcoitum
ENU: *N*-ethyl-*N*-nitrosourea
MEC: middle ear cavity
NTHi: nontypeable Haemophilus influenzae
OM: otitis media
SPF: specific pathogen-free
