# Supplementary material for: Mutation at the Evi1 Locus in Junbo Mice Causes Susceptibility to Otitis Media
Source: PLoS Genet. 2006 Oct 6;2(10):e149. doi: 10.1371/journal.pgen.0020149 (PMC1592239; doi:10.1371/journal.pgen.0020149)
Supplement: Supplementary file 1 [file pgen.0020149.st001.doc]

**Supplemental Table**

**T-dependent and T-independent responses in immune challenged wild-type and Junbo mice**

|  | **T-Dependent response** | | **T-Independent response** | |
| --- | --- | --- | --- | --- |
|  | (to KLH antigen) | | (to PPS antigen) | |
|  | **IgG1** | **IgG2a** | **IgM** | **IgG3** |
| **wild-type** | 47.97  (25.66 - 82.16) | 42.41  (6.13 – 184.30) | 9.16  (4.28 – 21.00) | 1.07  (0.45 – 2.14) |
| ***Jbo/+*** | 58.70  (28.02 –123.84) | 52.13  (24.18 – 79.69) | 12.82  (6.97 – 25.89) | 3.37  (0.36 – 9.00) |
| ***P*** | *0.307* | *0.101* | *0.096* | *0.064* |

Figures in the body of the table are means (and range) of relative responses for 42-56DAB n=10 wild-type, n= 10 *Jbo/+*

The Wilcoxon sum-of-ranks test was used to test for statistical differences.
